# Comparison of gait analysis before and after unilateral total knee arthroplasty for knee osteoarthritis

**DOI:** 10.1186/s13018-024-04891-w

**Published:** 2024-08-27

**Authors:** Jun Fukui, Yasumoto Matsui, Takafumi Mizuno, Tsuyoshi Watanabe, Marie Takemura, Shinya Ishizuka, Shiro Imagama, Hidenori Arai

**Affiliations:** 1 Department of Orthopedic Surgery, Nagoya University Graduate School of Medicine, Nagoya, Aichi 466-8550 Japan; 2 Center for Frailty and Locomotive Syndrome, National Center for Geriatrics and Gerontology, Obu, Aichi 474-8511 Japan

**Keywords:** Knee osteoarthritis, Total knee arthroplasty, Unilateral gait, Gait analysis, Gait symmetry

## Abstract

**Background:**

Gait ability can be objectively assessed using gait analysis. Three-dimensional gait analysis, the most commonly used analytical method, has limitations, such as a prolonged examination, high system costs, and inconsistently reported gait symmetry in patients with knee osteoarthritis (OA). Therefore, we aimed to evaluate the gait symmetry and changes before and after unilateral total knee arthroplasty (TKA) using the Walkway analyzer, a sheet-type gait analyzer.

**Methods:**

The healthy group included 38 participants from the Locomotor Frailty and Sarcopenia Registry study with lower limb pain or Kellgren–Lawrence classification grade 3 or 4 OA. The OA group included 34 participants from the registry study who underwent unilateral TKA. The walking speed, step length, step width, cadence, stride time, stance time, swing phase time, double-limb support phase time, stride, step length, and step width were analyzed per side using the Walkway gait analyzer.

**Results:**

No significant differences between the right and left sides were observed in the healthy group. In the OA group, the time indices and stance phase (*p* = 0.011) and the double-limb support phase time (*p* = 0.039) were longer on the contralateral side and the swing phase was longer on the affected side (*p* = 0.004) pre-operatively. However, these differences disappeared post-operatively. There were no significant differences in the spatial indices. Thus, this study revealed that patients undergoing unilateral TKA had an asymmetric gait pre-operatively, with a time index compensating for the painful side, and an improved symmetric gait post-operatively.

**Conclusions:**

The Walkway analyzer employs a simple test that requires only walking; hence, it is expected to be used for objective evaluation in actual clinical practice.

## Background

Decreased walking speed is associated with an increased risk of cerebrovascular disease and reduced activities of daily living [1, 2]. Furthermore, decreased walking speed is considered a health indicator and death predictor in older persons [3–5]. The walking speed declines gradually from the age of 65 years [6, 7]. However, certain comorbidities are considered risk factors for an early decline in walking speed [8, 9], particularly knee osteoarthritis (KOA), which is associated with reduced walking speed and is the most common cause of walking difficulties in older persons [10, 11]. The main symptom of KOA is pain during loading, and total knee arthroplasty (TKA) aims to improve this pain and the overall walking ability [12].

Gait ability can be objectively assessed using gait analysis. Over the years, gait analysis has mainly been performed using motion-capture three-dimensional (3D) cameras. However, the limitations of 3D gait analysis include a prolonged examination and high system costs [13]. Additionally, gait is originally considered symmetrical [14]; therefore, 3D gait analysis is often limited to one leg because of time constraints and the effort required from the patient [15–18]. However, the reported gait symmetry of patients with KOA is inconsistent, thus remaining controversial [12, 19–21].

Herein, we analyzed gait symmetry using the Walkway analyzer (Walkway MW-1000, ANIMA Corporation, Tokyo, Japan)—a two-dimensional sheet-type gait analysis device. The device employs a clinical test that allows for simultaneous assessment of the left and right gait by having patients walk on a 6 m sheet without needing to wear a monitor. Therefore, this study aimed to compare the gait of healthy participants and patients with severe KOA (before and after TKA) using the Walkway analyzer and clarify their differences. The study also analyzed the gait differences between the contralateral and affected sides before and after TKA.

## Methods

### Participants

The Locomotor Frailty Sarcopenia Registry (LFSR) study aimed to accumulate data on locomotive syndrome, frailty, and sarcopenia and establish a disease registry clarifying the relationship between these conditions. This study included the LFSR study participants who consented to participate during their outpatient visit for frailty assessment.

**Fig. 1:**
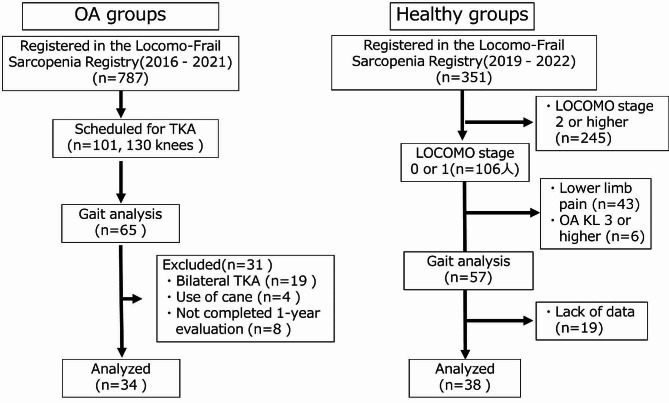
Study flowchart. Abbreviation: OA, osteoarthritis. TKA, total knee arthroplasty

One hundred and one patients (130 knees) from the 787 LFSR study participants scheduled for TKA between March 22, 2016 and July 26, 2021 consented to participate in this study. Sixty-five patients were eligible for pre-operative gait analysis. Among them, 19 participants who required bilateral TKA, 4 using canes, and 8 who could not be followed up for 1 year post-operatively were excluded (Fig. 1). Finally, the osteoarthritis (OA) group in this study included 34 participants (5 men and 29 women) from the LFSR study, with a mean age of 75.3 ± 6.8 years.

Regarding the healthy group, among the 351 LFSR study participants registered after April 2019, when full-length standing radiographs of the bilateral lower limbs were available, the following individuals were excluded: (1) 245 with Geriatric Locomotive Function Scale (LOCOMO) stage ≥ 2, (2) 43 with lower limb pain based on question three of the 25-question LOCOMO (LOCOMO-25 q03), (3) 6 with Kellgren–Lawrence (KL) grade ≥ 3 KOA, and (4) 19 with unavailable data. Individuals with evident hip or ankle OA were also excluded (Fig. 1). Finally, the healthy group consisted of 38 participants (26 men and 12 women), with a mean age of 74.7 ± 5.6 years.

### Inclusion criteria for the locomotor frailty sarcopenia registry study

The inclusion criteria for the LFSR study included the ability to (1) walk without support, (2) visit the hospital, (3) undergo examination 1 month pre-operatively and a 1-year follow-up after TKA, (4) receive a prior explanation of the study from a doctor and sign a consent form, and (5) participate in the study that included patients with a history of locomotive syndrome, frailty, or sarcopenia.

### Exclusion criteria for the locomotor frailty sarcopenia registry study

Patients meeting the following criteria were excluded from the LFSR study: patients (1) with visual or hearing impairments that interfered with daily life, (2) with paralysis at enrolment, (3) diagnosed with dementia (including patients taking dementia medication), (4) with neurological disorders and progressive limb dysfunction, (5) considered to be in the terminal phase, (6) who underwent simultaneous bilateral TKA, (7) who underwent TKA on the contralateral side within 1 year post-operatively, and (8) who used a cane during gait assessment after undergoing TKA.

### Gait analysis

Walking was measured using a 2.4 m long and 0.6 m wide sheet-type foot pressure-ground gait analyzer (Walkway MW-1000; ANIMA Corporation, Tokyo, Japan) on a 6.4 m long and 0.6 m wide walking path. The length of the walking path comprised the gait analyzer and 2 m front and rear aided paths. The participants walked twice at a comfortable speed, and the average values of the automatic measurements were used. Parameters measured for the gait analysis included basic gait information: walking speed (m/s), step length (cm), step width (cm), and cadence (steps/min); and unilateral gait information: stride time (s), stance time (s), swing phase time (s), double-limb support phase time (s), stride (cm), and step length (cm).

Basic gait information was compared between the healthy and pre-operative OA groups. Differences in the left and right unilateral gait information were compared in the healthy group. Additionally, pre-operative and post-operative basic and unilateral gait information were compared between the affected and contralateral sides in the OA group. Pain in the lower limb was graded based on LOCOMO-25 q03 as follows: (1) not painful or none (grade 0), (2) slightly painful or mild (grade 1), (3) moderately painful or moderate (grade 2), (4) significantly painful or severe (grade 3), and (5) severely painful or very severe (grade 4).

### Ethical considerations

This study was conducted with the approval of the Ethics Committee of the National Center for Geriatrics and Gerontology (date: December 3, 2015; study number: 881). Written informed consent was obtained from all the participants, and the study was conducted in accordance with the principles of the Declaration of Helsinki.

### Statistical analyses

Statistical analyses were performed using SPSS Statistics version 28.0 (IBM Corp., Armonk, NY, USA). Statistical significance was set at *p* < 0.05. Comparisons between the healthy and OA groups were performed using unpaired Student’s *t*-tests and the chi-square test. In contrast, comparisons within the OA group were performed using paired Student’s *t*-tests. Each *t*-test was subjected to a power analysis to estimate the power of the test.

## Results

Demographic data revealed no difference in the mean age between the healthy (74.7 years) and OA (75.2 years) groups. There were 24 men and 14 women in the healthy group and 5 men and 29 women in the OA group, with more women in the OA group (*p* < 0.0001). The mean statures were 159.8 cm and 150.9 cm in the healthy and OA groups, respectively. The mean body mass index (BMI) values were 23.2 kg/m^2^ and 26.4 kg/m^2^ in the healthy and OA groups, respectively. The OA group had a shorter stature (*p* < 0.0001) and higher BMI than the healthy group did (*p* < 0.0001). The LOCOMO grade was 0 and 1 in 2 and 36 patients in the healthy group, respectively, and 1, 2, and 3 in 3, 3, and 28 patients in the OA group, respectively.

Thirty-eight patients in the healthy group had KL grades 0–2 KOA, and 8 and 26 patients in the OA group had KL grades 3 and 4 KOA, respectively. Based on LOCOMO-25 q03, 38 patients in the healthy group experienced grade 0 pain. Conversely, eight, nine, eight, seven, and two patients in the OA group had LOCOMO-25 q03 grades 0, 1, 2, 3, and 4 pain, respectively. The OA group had more advanced LOCOMO and KL grades and experienced more pain than the healthy group did (*p* < 0.0001; Table 1).

**Table 1:** Comparison of participant characteristics between groups

		Healthy group	OA group	*P*
Age, y^a^		74.7 (72.8, 76.5)	75.2 (72.7, 77.6)	0.650
Sex^b^	Male	26 (68.4)	5 (14.7)	<0.0001
	Female	12 (31.6)	29 (85.3)	
Height (m) ^a^		159.8 (157.3, 162.3)	150.9 (148.4, 153.5)	<0.0001
BMI (kg/m^2^) ^a^		23.2 (22.2, 24.1)	26.4 (25.0, 27.7)	<0.0001
LOCOMO stage^b^	0	2 (5.6)	0	<0.0001
	1	36 (94.3)	3 (8.8)	
	2	0	3 (8.8)	
	3	0	28 (82.3)	
KL classification^b^	0–2	38 (100)	0	<0.0001
	3	0	8 (23.5)	
	4	0	26 (76.5)	
Pain^b^	0	38 (100)	8 (23.5)	<0.0001
	1	0	9 (26.5)	
	2	0	8 (23.5)	
	3	0	7 (20.6)	
	4	0	2 (5.9)	

^a^Data are presented as 95% confidence intervals

^b^Data are presented as n (%)

BMI, body mass index; KL, Kellgren–Lawrence classification (average of left and right); LOCOMO, Geriatric Locomotive Function Scale; OA, osteoarthritis

Pain, pain in the lower limb was rated as follows: not painful, none = 0; slightly painful, mild = 1; moderately painful, moderate = 2; significantly painful, severe = 3; and severely painful, very severe = 4 [based on question three of the 25-item LOCOMO].

**Fig. 2:**
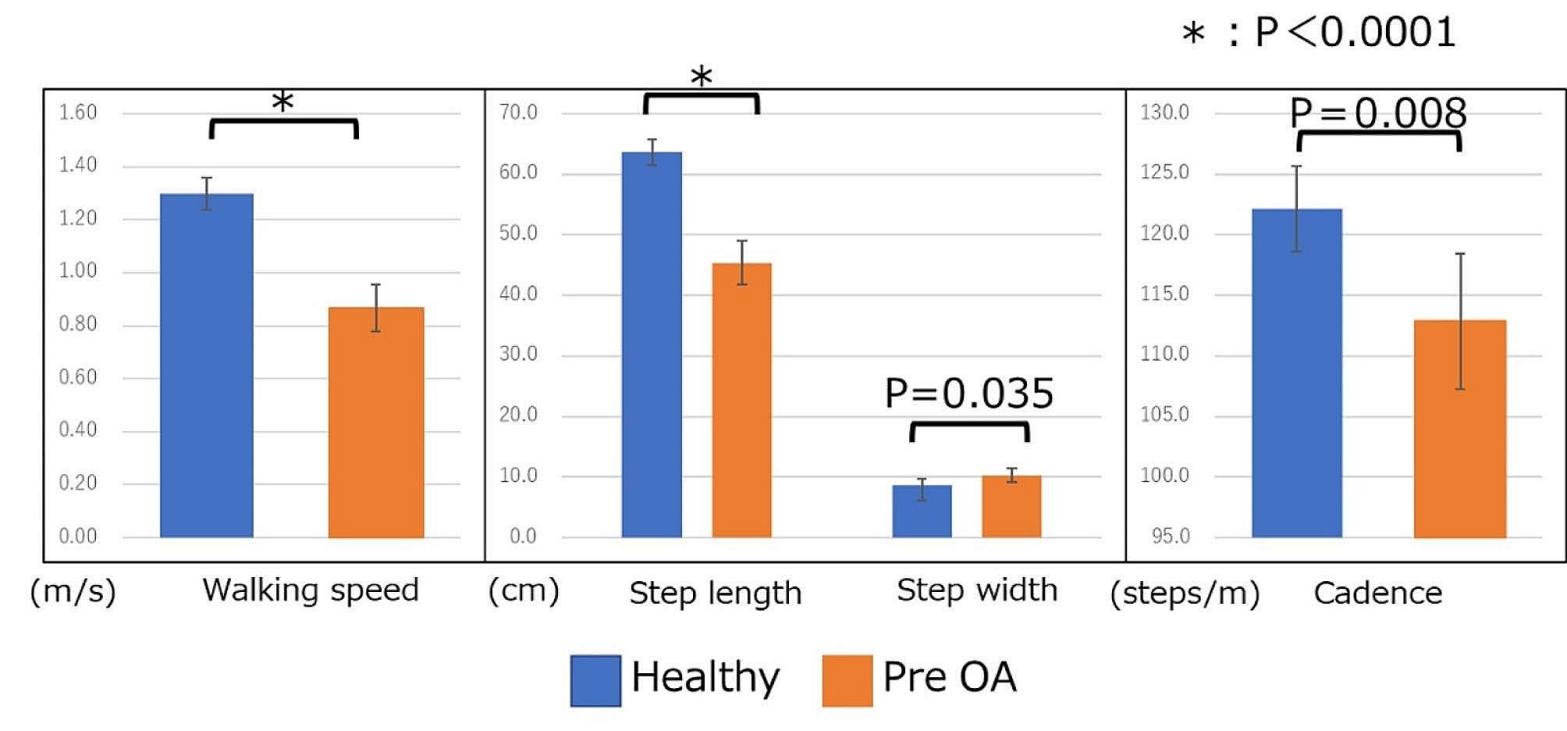
Basic gait information: healthy and pre-operative OA groups. Error bars indicate 95% confidence intervals. *significant difference between groups *p* < 0.0001. Healthy = healthy group, Pre-OA = pre-operative OA group

The mean walking speeds of the healthy and OA groups were 1.30 m/s (95% confidence interval [CI]: 1.24, 1.36 m/s) and 0.87 m/s (95% CI: 0.78, 0.95 m/s) (*p* < 0.0001), respectively, indicating that the healthy group was faster than the OA group was. Furthermore, the step lengths, a component of walking speed, were 63.7 cm (95% CI: 61.6, 65.7 cm) and 45.4 cm (95% CI: 41.8, 49.0 cm) in the healthy and OA groups (*p* < 0.0001), respectively. The step widths were 8.72 cm (95% CI: 7.75, 9.68 cm) and 10.3 cm (95% CI: 9.10, 11.50 cm) in the healthy and OA groups, respectively (*p* = 0.035). Additionally, the cadences were 122.2 steps/min (95% CI: 118.7, 125.7 steps/min) and 112.9 steps/min (95% CI: 107.3, 118.5 steps/min) in the healthy and OA groups (*p* < 0.0008), respectively. All measured parameters were better in the healthy group than in the OA group (Fig. 2). The power of the unpaired Student’s *t*-test was 1.00.

**Fig. 3:**
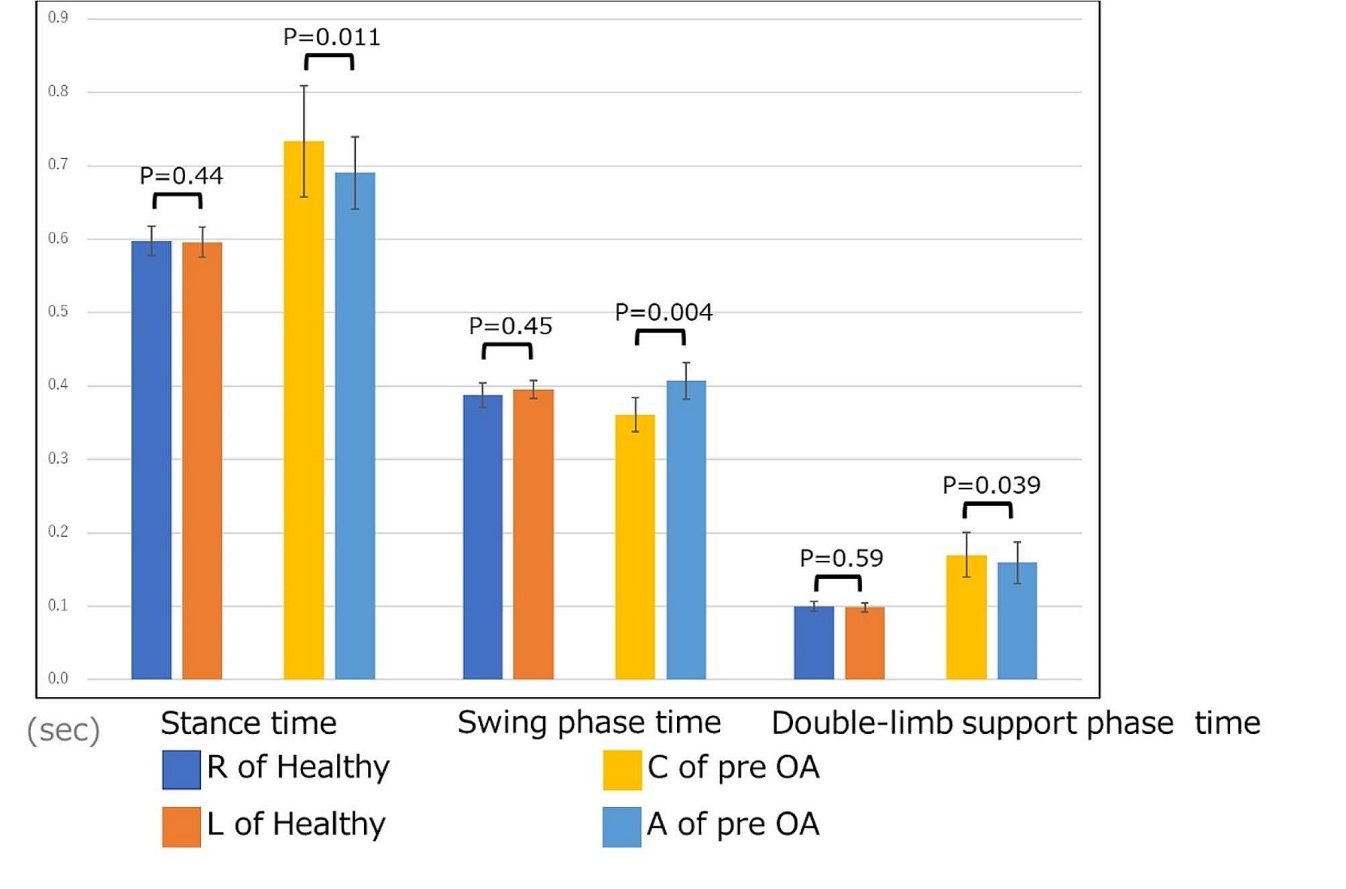
Unilateral gait information: healthy group (right and left) and OA group (contralateral and affected sides). R of Healthy = Right side of the healthy group, L of Healthy = Left side of the healthy group, C of pre-OA = Contralateral side of the pre-operative OA group, A of pre-OA = affected side of the pre-operative OA group

No differences were observed in the unilateral gait information between the left and right sides of the healthy group (Fig. 3). A pre-operative comparison of the contralateral and affected sides revealed that the stance time was longer on the contralateral side than on the affected side: 0.73 s (95% CI: 0.66, 0.81 s) and 0.69 s (95% CI: 0.64, 0.74 s), respectively (*p* = 0.011). The swing phase times were 0.36 s (95% CI: 0.34, 0.38 s) and 0.41 s (95% CI: 0.38, 0.43 s) on the contralateral and affected sides, respectively (*p* = 0.004). The double-limb support phase time was significantly longer on the contralateral side than on the affected side (0.17 s [95% CI: 0.14, 0.20 s] and 0.16 s [95% CI: 0.13, 0.19 s], respectively [*p* = 0.039; Fig. 3]). No significant differences were observed in the spatial indices (Fig. 3).

**Fig. 4:**
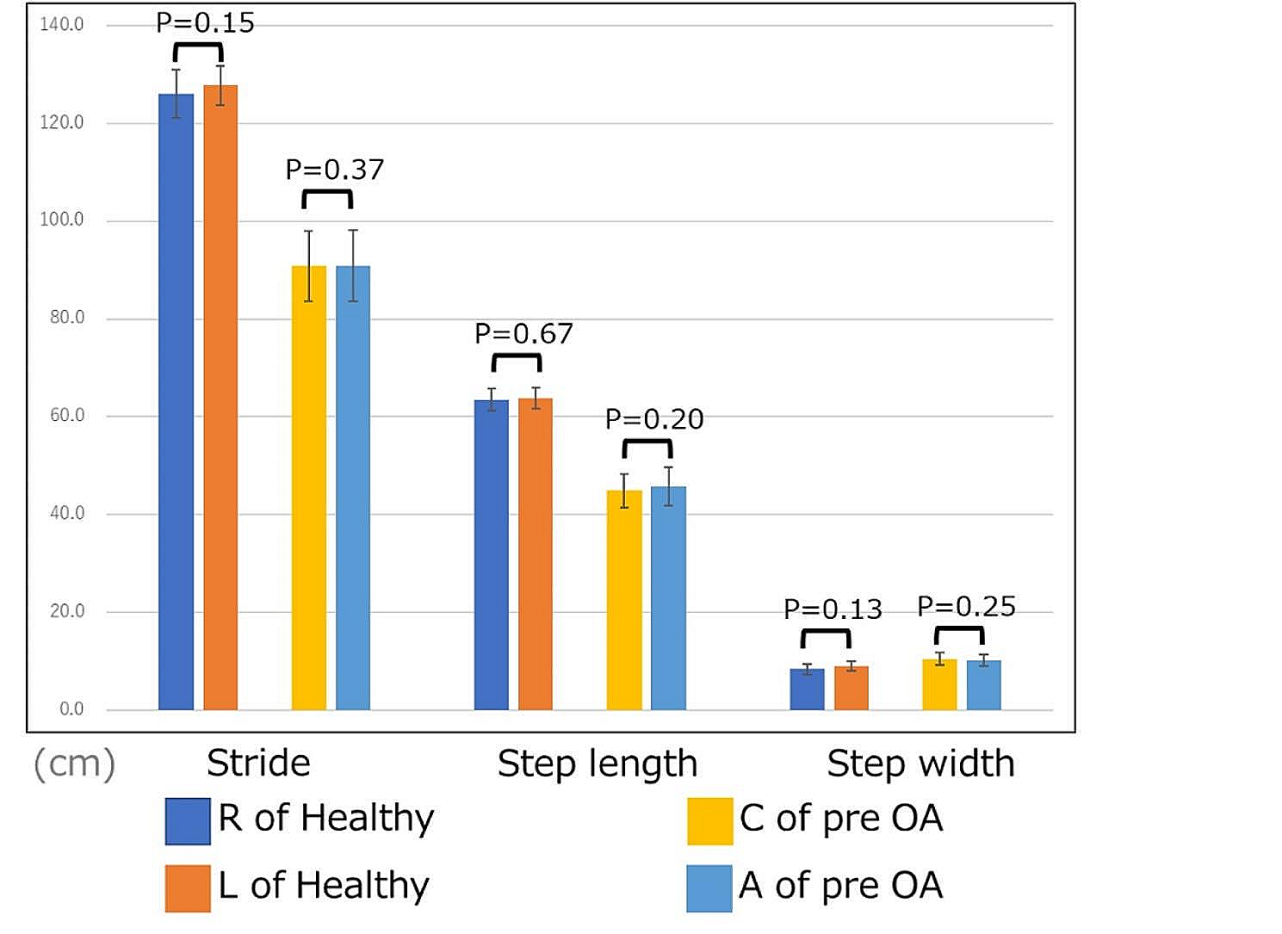
Basic gait information (changes after TKA): pre-operative OA, post-operative OA, and healthy groups. Post-OA = post-operative OA group

Significant improvements were observed in the basic gait information of the OA group pre-operatively and post-operatively as follows: (1) gait speed, 0.87 m/s (95% CI: 0.78, 0.95 m/s) and 1.08 m/s (95% CI: 1.00, 1.16 m/s) (*p* < 0.0001); (2) step length, 45.4 cm (95% CI: 41.8, 49.0 cm) and 52.6 cm (95% CI: 49.8, 55.5 cm) (*p* < 0.0001); and (3) cadence, 112.9 steps/min (95% CI: 107.3, 118.5 steps/min) and 122.8 steps/min (95% CI: 117.4, 128.2 steps/min) (*p* = 0.0003), respectively. No significant improvement was observed in the step width post-operatively. Post-operative comparison of the healthy and OA groups revealed an improvement in the step length and cadence, although no significant differences were observed between the groups. In addition, the walking speed and step length continued to be better in the healthy group than in the OA group (*p* < 0.0001; Fig. 4). The power of the paired Student’s *t*-test was 0.96.

**Fig. 5:**
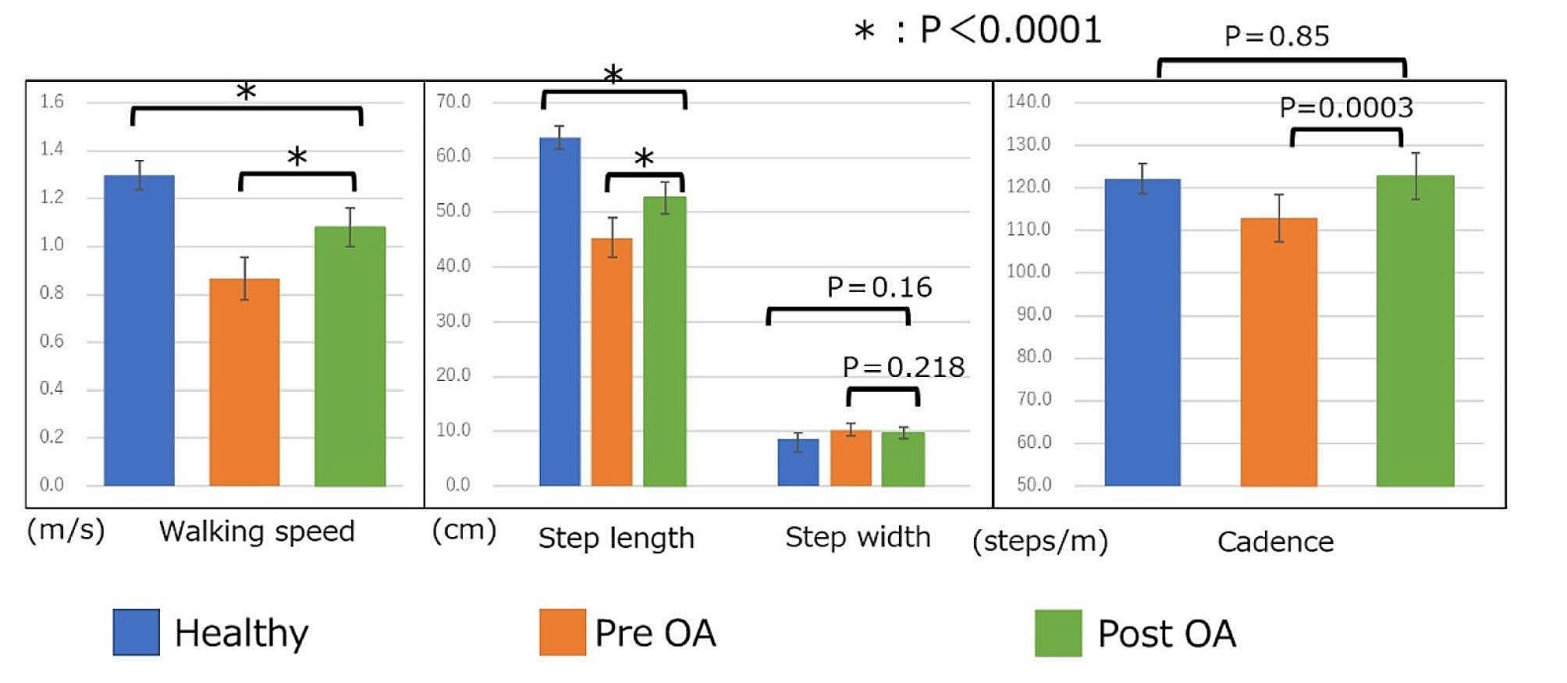
Unilateral gait information: contralateral and affected sides of both the pre-operative and post-operative OA groups. C of pre-OA = contralateral side of the pre-operative OA group, A of pre-OA = affected side of the pre-operative OA group; C of post-OA = contralateral side of the post-operative OA group, A of post-OA = affected side of the post-operative OA group

The following significant improvements in unilateral gait information of the OA group were observed on comparing the pre-operative and post-operative values on the contralateral side: (1) stride time, 1.09 s (95% CI: 1.02, 1.16 s) and 1.00 s (95% CI: 0.95, 1.04 s) (*p* = 0.003); (2) stance time, 0.73 s (95% CI: 0.66, 0.81 s) and 0.62 s (95% CI: 0.59, 0.65 s) (*p* = 0.003); (3) double-limb support time, 0.17 s (95% CI: 0.14, 0.20 s) and 0.12 s (95% CI: 0.11, 0.13 s) (*p* = 0.004); (4) stride, 91.0 cm (95% CI: 83.6, 98.1 cm) and 105.6 cm (95% CI: 99.7, 110.9 cm) (*p* = 0.0002); and (5) step length, 44.9 cm (95% CI: 41.5, 48.4 cm) and 52.7 cm (95% CI: 49.8, 55.6 cm) (*p* < 0.0001), respectively (Fig. 5).

**Fig. 6:**
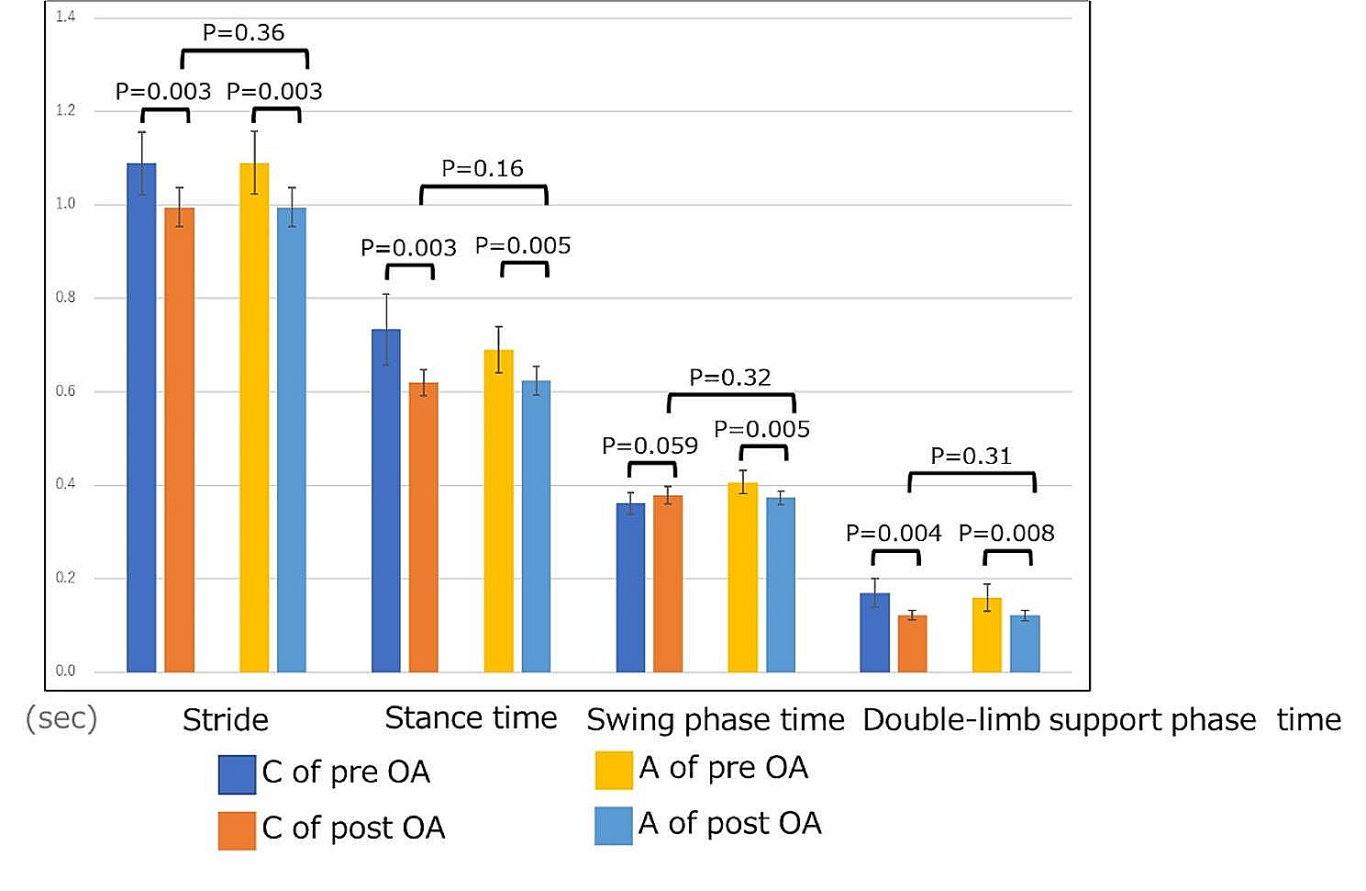
Unilateral gait information (time indices): contralateral and affected sides of both preoperative and postoperative OA groups

**Fig. 7:**
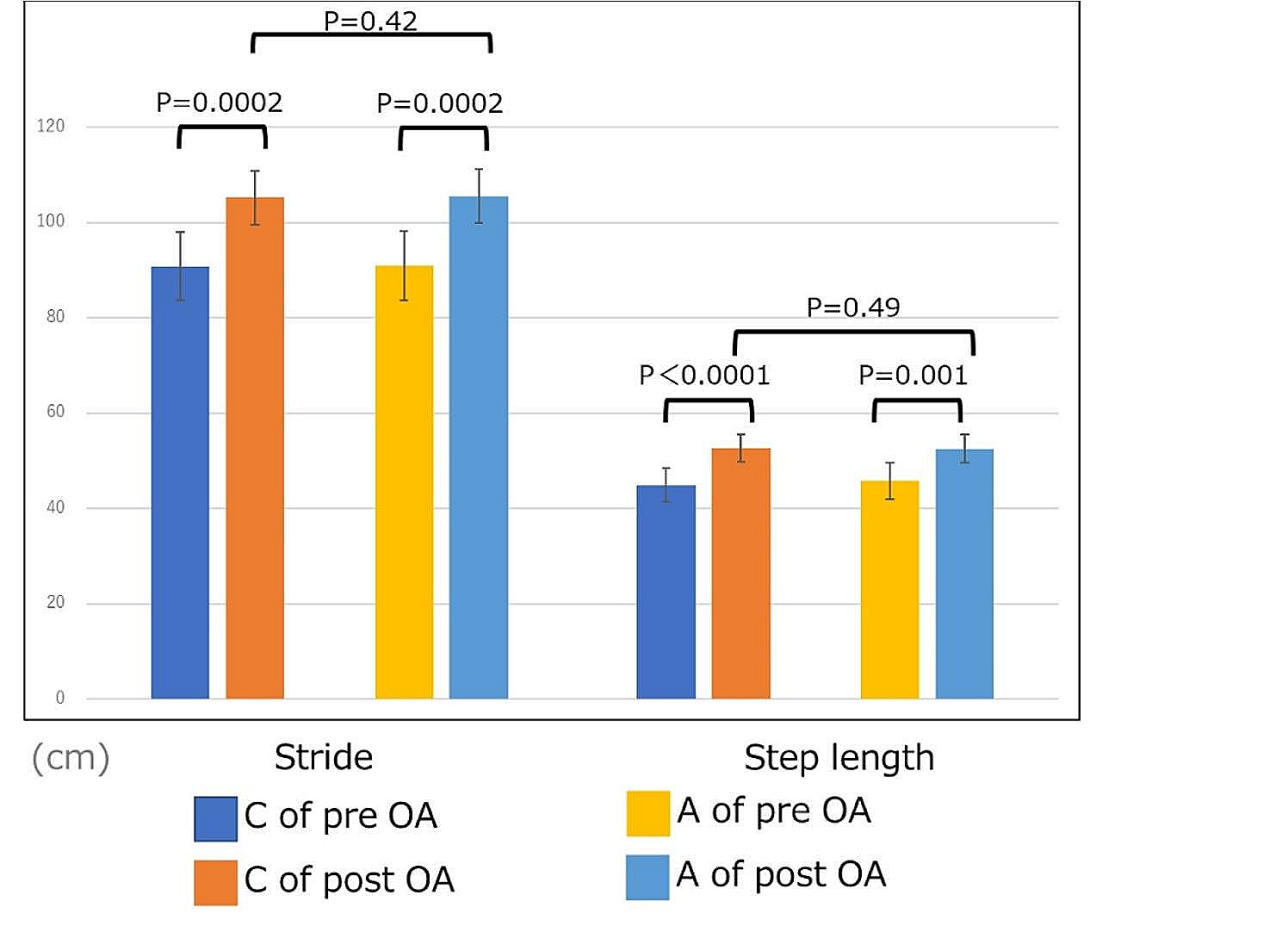
Unilateral gait information (spatial time indices): contralateral and affected sides of both preoperative and postoperative OA groups

Significant improvements observed on comparing the pre-operative and post-operative values of the affected side included the following: (1) stride time, 1.09 s (95% CI: 1.02, 1.16 s) and 1.00 s (95% CI: 0.95, 1.04 s) (*p* = 0.003); (2) stance time, 0.69 s (95% CI: 0.64, 0.74 s) and 0.62 s (95% CI: 0.59, 0.66 s) (*p* = 0.005); (3) swing phase, 0.41 s (95% CI: 0.38, 0.43 s) and 0.37 s (95% CI: 0.36, 0.39 s) (*p* = 0.005); (4) double-limb support time, 0.16 s (95% CI: 0.13, 0.19 s) and 0.12 s (95% CI: 0.11, 0.13 s) (*p* = 0.008); (5) stride, 91.0 cm (95% CI: 83.8, 98.2 cm) and 105.6 cm (95% CI: 99.9, 111.3 cm) (*p* = 0.0002); and (6) step length, 45.8 cm (95% CI: 41.9, 49.7 cm) and 52.6 cm (95% CI: 49.7, 55.5 cm) (*p* = 0.001), respectively. The significant pre-operative differences observed between the contralateral and affected sides were nonexistent after TKA (Figs. 6 and 7).

## Discussion

The lack of differences in the left and right unilateral gait information of the healthy group was consistent with the findings of previous reports [12, 22]. Studies have reported that the walking speed and cadence decrease and step length shortens in patients with KOA [11, 23–26]. In this study, the pre-operative results in the OA group were similar to those in previous studies. The observed decrease in cadence in the OA group can be attributed to a prolonged gait cycle time—including the free and stance phases. The stance and swing phases constitute 60% and 40% of the gait cycle, respectively [27, 28]. When the walking speed decreases, the duration of the double-limb support phase within the stance phase is prolonged, and the swing phase is shortened.

A longer double-limb support time indicates that both feet are on the ground simultaneously, which is highly stable and convenient for preventing falls in older persons. Studies on KOA report that the proportion of the double-limb support phase increases with decreasing gait speed [11, 24, 25], similar to the results of this study. Pre-operatively, the stance and double-limb support phases were shorter on the affected side and the swing phase was longer. This suggests that the gait may have compensated for pain on the affected side. Additionally, the gait cycle in each leg before unilateral TKA was different, indicating asymmetric gait.

KOA is often present bilaterally and, in 80% of cases, radiographically diagnosed unilateral KOA leads to bilateral KOA after 12 years [29]. However, symptoms and OA progression may vary significantly within an individual at different times. Previous reports of gait asymmetry in patients with unilateral KOA have been within the scope of biomechanics [12, 19], with no reported differences in the temporal or spatial indices [19–21]. Furthermore, gait analysis based on the KL classification alone may be insufficient because simple radiographic findings may not correspond with the pain grade [30]. Additionally, a strong correlation between pain and the length of the swing phase has been reported [31].

This study differed from previous studies on KOA because it did not assess pain and included patients with mild-to-moderate OA (KL grades 2–3) [19–21]. The OA group included patients with severe OA (KL grade 3 or 4). The difference between the healthy and affected sides was assumed to result from patients experiencing the most pain before unilateral TKA surgery. Additionally, a study reported that the swing phase length determined the pain level, function, and quality of life of patients with KOA [31]. Hence, if objective asymmetry can be confirmed via walking, it could be a factor in future surgery consideration.

Furthermore, reduced walking speed, step length, and cadence can be improved by treating OA [32]. In this study, TKA resulted in similar improvements in these parameters. However, while the cadence improved to the same level in the OA group as in the healthy group, the step length did not improve. This may be because step length correlates with the strength of the quadriceps muscles [33], which is often affected by sarcopenia in patients with KOA [34]. Moreover, gait parameters after TKA reportedly improve between 1 and 2 years post-operatively [35]. Hence, in this study, participants’ muscle strength might not have fully improved at 1 year post-operatively. The gait cycle is said to shorten after TKA [36]. In this study, the gait cycle improved significantly on participants’ contralateral and affected sides. Additionally, the difference between the contralateral and affected sides was nonexistent after TKA.

Unlike questionnaires based on subjective data obtained from participants, gait analysis is based on objective data and used in conservative treatment, pre-operative and post-operative assessment, and rehabilitation therapy, regardless of subjective symptoms [37]. Currently, gait analysis in practice is mainly based on 3D analysis. However, despite its history dating back > 100 years [38, 39], it remains impractical owing to challenges such as the (1) requirement of a 30 min duration, (2) tedious data analysis, (3) strenuous effort required from the participant, (4) machine cost, and (5) need for a special examination room [13]. Therefore, introduction of modern and simplified methods is expected. The Walkway gait analyzer in this study employs a simple test requiring a few minutes with minimal burden on the participant. Additionally, it does not require a special laboratory.

No previous studies have compared the gait on the contralateral and affected sides before and after TKA using a sheet-type gait analyzer. Nevertheless, this study’s results using the Walkway analyzer were similar to those previously obtained using motion-capture gait analyzers. Hence, the Walkway analyzer could be considered a valid and reliable gait analyzer. Clearly, gait asymmetry appears in severe OA because of temporary factors. The Walkway analyzer, which enables data to be easily and simultaneously collected on both sides, is expected to aid the assessment of gait asymmetry in routine outpatient care. Furthermore, gait analysis using the Walkway analyzer can be an objective decision-making criterion when making surgical decisions considering the reduced walking function and quality of life of patients with OA.

The study has a few limitations. First, the study population included only Japanese participants, and a difference in the sex ratio and BMI existed between the healthy and OA groups. Second, pain on the contralateral and affected sides was not assessed separately in the OA group, and the contralateral side of the OA group could not be assessed using simple radiographs. The pain experienced by participants was assessed as bilateral lower limb pain, based on LOCOMO-25 q03. Hence, the assessment of pain in each leg will be required in the future. Third, the contralateral knee assessment was affected by the fact that bilateral lower-limb standing radiographs were not obtained before April 2019.

## Conclusion

The Walkway gait analyzer produced results similar to those previously reported using motion-capture gait analyzers. Patients who underwent unilateral TKA had an asymmetric gait pre-operatively, with shorter stances and double-limb support phases on the affected side and longer swing phases. Additionally, the gait cycle improved post-operatively on the contralateral and affected sides, resulting in a symmetric gait. In conclusion, the Walkway gait analyzer employs a simple test that requires a few minutes. Hence, it is a convenient and efficient assessment tool that can be introduced in real-life clinical practice.

## Data Availability

Due to the nature of this research, participants of this study did not agree for their data to be shared publicly, so supporting data is not available.

## Abbreviations

3D: Three-dimensional
BMI: Body mass index
CI: Confidence interval
KL: Kellgren–Lawrence
KOA: Knee osteoarthritis
LFSR: Locomotor Frailty Sarcopenia Registry
LOCOMO: Geriatric Locomotive Function Scale
LOCOMO-25 q03: Question three of the 25-question LOCOMO
OA: Osteoarthritis
TKA: Total knee arthroplasty
